# Anatomical Variations in the Posterior Tibial Slope in the North Indian Population: A Hospital-Based Study

**DOI:** 10.7759/cureus.41338

**Published:** 2023-07-03

**Authors:** Shailendra Singh, Anmol Chaurasia, Kumar Shantanu, Ravindra Mohan, Sripal Chaudhary, Deepak Kumar, Arpit Singh

**Affiliations:** 1 Department of Orthopaedic Surgery, King George's Medical University, Lucknow, IND

**Keywords:** anatomical variation, posterior tibial slope, north indian population, lateral knee radiographs, knee

## Abstract

Background: The measurement of the posterior tibial slope (PTS) angle is crucial for various knee surgeries such as total knee replacement, high tibial osteotomy, and anterior cruciate ligament reconstruction. This hospital-based study aimed to determine the average PTS angle in the knee joints of the North Indian population and provided valuable data to aid knee surgeries in this region.

Methods: An analysis of 200 plain X-ray films, specifically the true lateral view of both knees in 20-degree flexion, was conducted on participants who exhibited skeletal maturity with no arthritis, tumours, or previous knee surgeries. The PTS measurements were performed manually. The data were analyzed statistically by matching them with variables such as gender, age, laterality, and body mass index (BMI).

Results: The study revealed the following findings for the posterior tibial slope in a section of the North Indian population: there was no significant laterality difference (right knee: 12.76±2.35°, left knee: 12.55±2.46°); no notable sexual dimorphism (males: right knee - 12.79±2.65°, left knee - 12.25±2.65°, females: right knee - 12.73±2.11°, left knee - 12.77±2.30°). However, as age advanced, there were significant differences observed (PTS: 14.27±1.28° and 13.84±1.80° in the 18-40 years age group, 11.36±1.76° and 11.31±1.97° in the 41-60 years age group, 10.32±2.82° and 10.56±3.04° in the >60 years age group for the right and left knee, respectively). No significant correlation was found with BMI (PTS: 13.12±3.13° and 12.59±3.14° for BMI <25, 12.88±2.15° and 12.80±2.34° for BMI 25-30, 12.00±2.09° and 11.66±2.99° for BMI >30 in the right and left knee, respectively).

Conclusions: The study demonstrated significant variations in the posterior tibial slope based on age, emphasizing the need for individualized treatment in knee surgeries. The research provided valuable insights into normal PTS values specific to the North Indian population, offering regional data to inform knee surgery procedures.

## Introduction

The posterior tibial slope (PTS) plays a critical role in the stability and biomechanics of the knee joint. Changes in the PTS angle can impact knee function and are associated with various knee pathologies, including anterior cruciate ligament (ACL) rupture and knee instability. For example, there is an increased risk of ACL tear in the presence of an increased PTS angle, which indicates a positive correlation between PTS angle and ACL injury. Patients with increased PTS have a higher risk of developing anterior tibial translation [1]. Moreover, the PTS angle influences surgical considerations such as high tibial osteotomies and knee arthroplasty. With increasing PTS, range of motion (ROM) and activity levels increase significantly. When the increase in PTS reaches a certain angle, the impact on ROM and activity level plateaus [2,3].

While the importance of the PTS angle is well recognised, there is a lack of specific data regarding its normal range in the North Indian population. Ethnicity has been shown to influence the standard value of the PTS angle, highlighting the need for population-specific studies. Prior research has demonstrated variations in the PTS angle across different ethnic groups, with values ranging from 11.9° in Igbo individuals of South East Nigeria to 14.7° in Chinese individuals and 14.1° and 12.5° among female and male Pakistani adults, respectively [4-6].

Accurate measurement and understanding of the PTS angle are crucial for optimising surgical outcomes and designing prostheses that align with the anatomical tibial slope. Additionally, the PTS angle impacts knee joint stability and the forces experienced by the cruciate ligaments [7]. A posteriorly elevated PTS angle increases the risk of ACL injury, while a reduced angle may be protective in ACL-deficient knees [8].

The primary objective of this study was to determine the mean PTS angle in knee joints in the North Indian population. By analysing plain X-ray films obtained from individuals without arthritis, tumours, or prior knee surgery, we aimed to provide valuable data to guide knee surgeries in this region. The findings of this study will contribute to enhancing surgical planning, implant selection, and postoperative outcomes in North Indian patients.

Understanding the regional anatomical variation of the PTS angle will help orthopaedic surgeons tailor their approaches and techniques to the specific needs of the North Indian population. This study will provide essential insights into the normal PTS values in this demographic, contributing to evidence-based guidelines for knee surgeries and optimising patient care.

## Materials and methods

The study was conducted at the Department of Orthopaedics, King George's Medical University (KGMU), Lucknow, India. The study was approved by the Institutional Ethics Committee of King George's Medical University (approval number: XII-PGTSC-IIA/P9). Informed consent was obtained from each patient after explaining the procedures and tests in their native language.

Patients who exhibited pathologies that could alter the normal architecture of the knee joint or had congenital abnormalities or deformities affecting the knee joint or lower leg, which could disrupt the alignment of the posterior tibial slope (PTS), were excluded from the study. Additionally, patients with tumours, previous knee surgery, diagnosed ACL injuries, advanced osteoarthritis (assessed using Kellgren-Lawrence grading [9]), or those who did not provide consent were excluded.

All patients visiting the outpatient department (OPD) for individual symptoms underwent radiological examinations based on their clinical presentation. A comprehensive medical history and standard clinical examination were performed on each patient to rule out ligament problems, with a particular focus on ACL damage assessed through a combination of tests including the anterior drawer test, Lachman test, and pivot shift test [7].

Anteroposterior and true lateral view radiographs, showing superimposition of the femoral condyles, were obtained for the selected subjects by experienced technicians using the same X-ray machine in the department of radiodiagnosis at KGMU. The radiographic technique was standardised for all subjects. For the lateral view radiograph, patients were positioned on the same side of the affected knee, flexing the knee approximately 25°-30°. The central X-ray beam was directed vertically towards the medial aspect of the knee joint, with a cephalad angulation of about 5°-7° [10].

**Figure 1:**
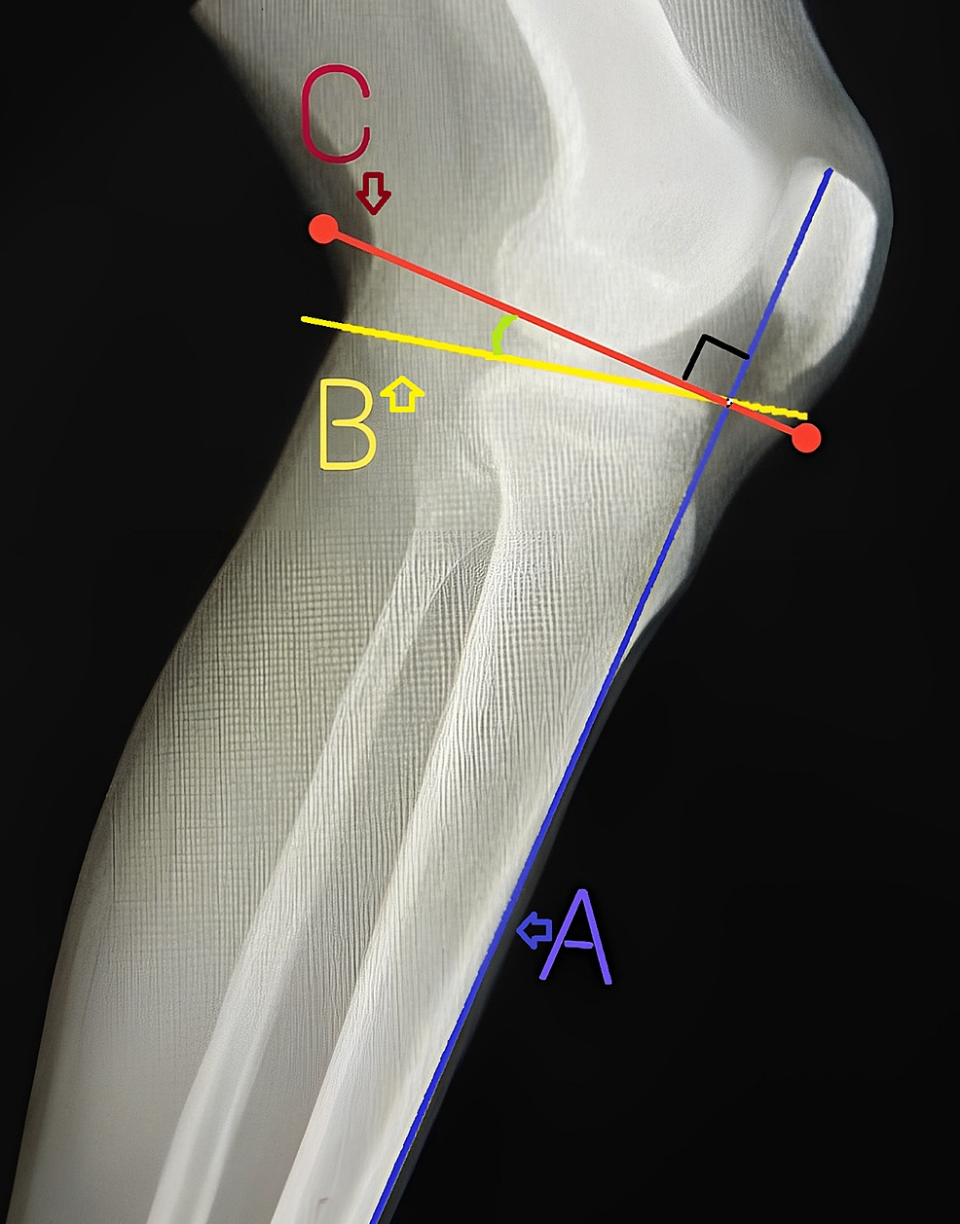
An X-ray of the knee joint (lateral view) showing the measurement of the posterior tibial slope (PTS) A: anterior cortical line; B: line tangential to the tibial plateau; C: line perpendicular to A at the point of intersection of A and B

**Figure 2:**
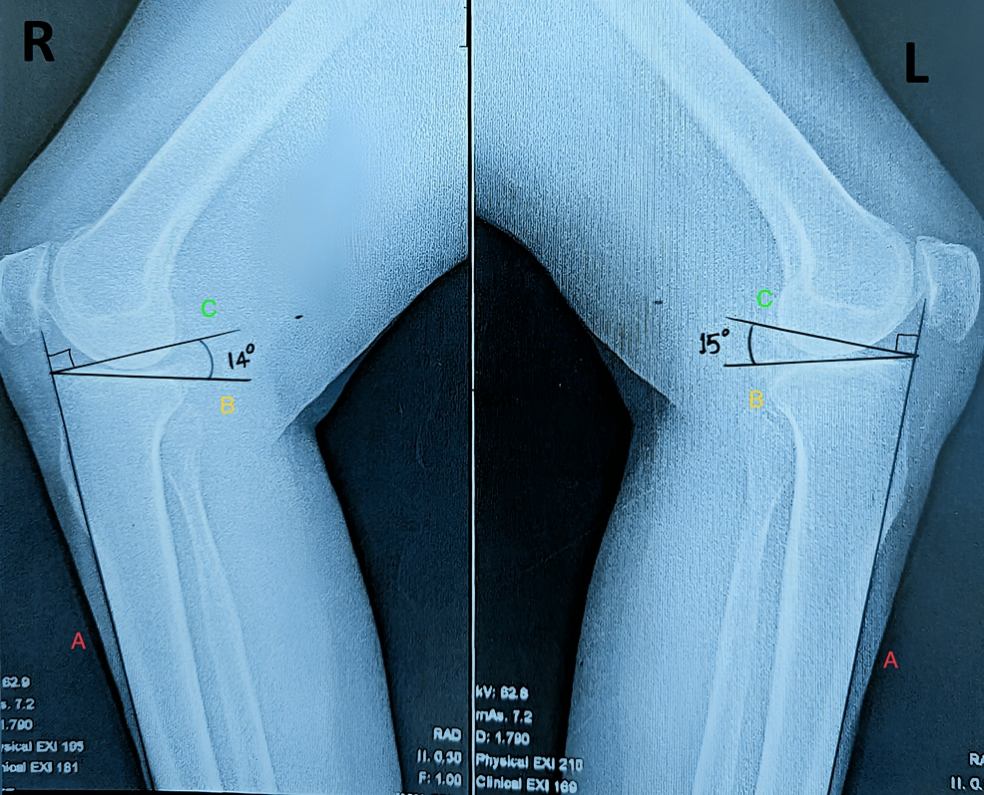
X-rays of the right (R) and left (L) knee joints (lateral views) showing the measurement of the posterior tibial slope (PTS) A: anterior cortical line; B: line tangential to the tibial plateau; C: line perpendicular to A at the point of intersection of A and B PTS of the (R) and (L) knees are 14° and 15°, respectively.

The measurement of the posterior tibial slope (PTS) angle was performed manually. A straight line (anterior cortical line) was drawn along the anterior cortex of the middle of the shaft of the tibia, and it was extended proximally to be intersected by the second straight line drawn tangential to the proximal tibial articular surface connecting the anterior and posterior ends of the tibial plateau. A further straight line was drawn from the point of intersection, perpendicular to the anterior cortical line. The angle between this perpendicular line and the tangential line along the tibial plateau is the PTS (Figures 1-2).

It is important to note that the measured angle represented a two-dimensional approximation of the complex, asymmetric three-dimensional surface of the medial and lateral plateaus. This method was based on the description by Moore and Harvey [11].

Radiographs showing osteophytes in the anterior and/or posterior ends of the tibial plateau were excluded from the measurement process due to the difficulty in obtaining accurate measurements.

By adhering to these standardised methods and exclusion criteria, the study aimed to ensure accurate and consistent measurement of the PTS angle in the study population.

## Results

The study investigated the posterior tibial slope (PTS) in a section of the North Indian population. The PTS angles were measured on both the right and left knees of the participants. The mean PTS angles (± standard deviation) for the right knee were 12.76°±2.35°, and for the left knee, they were 12.55°±2.46° (Table 1).

**Table 1:** Posterior tibial slope (°) mean values, SDs, and ranges of distribution for the left and right knees

Posterior tibial slope	Mean±SD	Minimum	Maximum
Right knee	12.76±2.35	6.0	24
Left knee	12.55±2.46	5	22

No significant differences were observed between the right and left knees, indicating no laterality.

Sexual dimorphism analysis revealed no significant differences in PTS angles between males and females. The mean PTS angles for males in the right knee were 12.79°±2.65°, and for the left knee, they were 12.25°±2.65°. The mean PTS angles for females in the right knee were 12.73°±2.11°, and for the left knee, they were 12.77°±2.30° (Table 2).

**Table 2:** Gender distribution of the posterior tibial slope measurements

Gender	Right knee	Left knee
Male	12.79±2.65	12.25±2.65
Female	12.73±2.11	12.77±2.30
F-value	0.040	2.178
P-value	0.841	0.142

Age-related differences in PTS angles were observed. As age advanced, a significant decrease in PTS angles was found. In the 18-40 age group, the mean PTS angles for the right knee were 14.27°±1.28°, and for the left knee, they were 13.84° ± 1.80°. In the 41-60 age group, the mean PTS angles for the right knee were 11.36°±1.76°, and for the left knee, they were 11.31°±1.97°. In the above-60 age group, the mean PTS angles for the right knee were 10.32°±2.82°, and for the left knee, they were 10.56°±3.04° (Table 3).

**Table 3:** Comparison of the posterior tibial slope measurements of the right and left knees with respect to age

Age (in years)	Right knee	Left knee
18-40	14.27±1.28	13.84±1.80
41-60	11.36±1.76	11.31±1.97
>60	10.32±2.82	10.56±3.04
F-value	89.763	45.245
P-value	<0.001	<0.001

Body mass index (BMI) also demonstrated a significant correlation with PTS angles. In participants with a BMI <25, the mean PTS angles for the right knee were 13.12°±3.13°, and for the left knee, they were 12.59°±3.14°. In participants with a BMI between 25 and 30, the mean PTS angles for the right knee were 12.88°±2.15°, and for the left knee, they were 12.80°±2.34°. In participants with a BMI >30, the mean PTS angles for the right knee were 12.00°±2.09°, and for the left knee, they were 11.66°±2.99° (Table 4).

**Table 4:** Posterior tibial slope distribution on the basis of BMI

Body mass index	Right knee	Left knee
<25	13.12±3.13	12.59±3.14
25-30	12.88±2.15	12.80±2.34
>30	12.00±2.09	11.66±2.99
F-value	11.105	9.098
P-value	0.001	0.001

## Discussion

In the present study, we investigated the posterior tibial slope (PTS) in a sample of 200 patients from the North Indian population. The majority of the participants (52.5%) belonged to the 18-40 age group, followed by 35.0% in the 41-60 age group, and 12.5% were over 60 years of age, with a mean age of 44.02±13.68 years. The study population exhibited a male predominance (56.5%). Additionally, 54.0% of patients had a BMI less than 25 kg/m², 36.5% had a BMI between 25 and 30 kg/m², and only 19.5% had a BMI greater than 30 kg/m², with a mean BMI of 27.67±2.67 kg/m².

Our findings were consistent with previous studies. Medda et al. reported similar mean ages for males and females (46 years and 44 years, respectively), with no significant difference between the left and right knees (P>0.05) [12]. Nekkanti et al. also found no significant difference in posterior tibial slope between genders, with a similar mean age for the male and female participants [13].

Moore and Harvey analysed the slope in the American population and reported a range of 7°-22°, with a mean and standard deviation of 14° and 3.7°, respectively [11]. Our study's findings (range 6°-24°, mean SD 13.6°-3.5°) were comparable to the results of Moore and Harvey's in a similar study [11]. Brazier et al. investigated the French population and found a smaller range (3.47°-20.29°) and mean ± SD (11.4°±3.6°) [14]. Genin et al. also reported lower values in the French population, ranging from -1° to 18°, with a mean±SD of 7.9°±3.2° [15]. Chiu et al. studied the Chinese population and found similar results to ours, with a range of 5°-22° and a mean ± SD of 14.7°±3.6° [5]. In the Nigerian population, Didia and Jaja reported a wider range (0°-24°) and a comparable mean±SD (12.3°±4.9°) [16].

Regarding the association of posterior tibial slope with age, our study found significant differences as age advanced, with lower PTS angles in older age groups (P< 0.05). These findings emphasise the need for individualised treatment in knee surgery, considering age-related variations in PTS.

We also investigated the association of the posterior tibial slope with gender and BMI. Our results revealed no significant differences between males and females, indicating a lack of sexual dimorphism in the posterior tibial slope. However, we found a significant t-correlation between BMI and posterior tibial slope, with higher BMI categories associated with lower PTS angles (p < 0.05).

Limitations of the study

Our study was conducted at a single centre in North India, and this is the potential limitation of the present study. For a better understanding of anatomical variations in PTS, a larger multicentric study should be conducted.

## Conclusions

Our study examined the posterior tibial slope (PTS) in 200 patients from the North Indian population. Age and BMI were identified as significant factors associated with PTS variations. The findings emphasise the need for individualised treatment approaches in knee surgery, considering age-related differences in PTS. Additionally, we observed a correlation between BMI and PTS, highlighting the influence of body weight on knee biomechanics.

Our study provides valuable regional data on normal PTS values in the North Indian population, aiding in surgical planning and improving outcomes. Further research is needed to explore the implications of PTS variations on knee joint stability and pathologies. Overall, this knowledge enhances evidence-based practice and personalised care in knee surgery.
